# Positive bias for European men in *peer reviewed *applications for faculty position at Karolinska Institutet

**DOI:** 10.12688/f1000research.13030.2

**Published:** 2018-08-14

**Authors:** Sarah Holst, Sara Hägg

**Affiliations:** 1 Department of Neuroscience, Karolinska Institutet, Stockholm, Sweden; 2 Department of Medical Epidemiology and Biostatistics, Karolinska Institutet, Stockholm, Sweden

**Keywords:** equality, diversity, life science, peer review, bibliometry, faculty positions, multivariable analysis, principal component analysis

## Abstract

**Background:** Sweden is viewed as an egalitarian country, still most of the professors are Swedish and only 25% are women. Research competence is evaluated using *peer review*, **which is regarded as an objective measure in the meritocracy system. Here we update the investigation by Wold & Wennerås (1997) on women researcher’s success rate for obtaining a faculty position, by examining factors (gender, nationality, productivity, etc.) in applications for an Assistant Professorship in 2014 at Karolinska Institutet.

**Methods:** Fifty-six applications, 26 Swedish and 21 women applicants, were scored both on merits and projects by six external reviewers. Additional variables, including grants and academic age, calculated as the number of years since PhD excluding parental or sick leave, were gathered. Productivity was assessed by calculating a composite bibliometric score based on six factors (citations, publications, first/last authorships, H-index, high impact publication).

**Results: **Overall, academic age was negatively correlated with scores on merits, as assessed by *peer review*, although not reaching statistical significance. In men, associations between scores on merits and productivity ( *P*-value=0.0004), as well as having received grants ( *P*-value=0.009) were seen. No associations were found for women. Moreover, applicants with a background from the Middle East were un-proportionally found in the lowest quartile (Fisher exact test *P*-value=0.007).

**Conclusions:** In summary, the gender inequality shown in *peer review* processes in Sweden 20 years ago still exists. Furthermore, a bias for ethnicity was found. In order to keep the best scientific competence in academia, more efforts are needed to avoid selection bias in assessments to enable equal evaluations of all researchers.

## Introduction

The key to promoting innovative research is a career system based on scientific competence, often assessed by *peer review* based on feasibility, novelty and significance of a research project in combination with assessing the merits of the applicant, regardless of gender, sexuality, ethnicity, religion, disability or age. However, the *peer review* process has been shown to be subjected to substantial bias ^1– 3^. Hence, the system of meritocracy is rather enforcing than reducing inequality and contributes to the uneven distributions of gender and ethnicity in academia.

In 1997 Wennerås and Wold ^2^ concluded that women were less likely than men to be recruited to faculty positions in Sweden. Twenty years later, despite high standards in equality and diversity ^4^, only 25% of the professors at Swedish Universities are women, and 23% have an international background, in spite of more than 50% of the doctoral students being women or students with other nationalities ^5^. The increase of women professors is slow, and the Swedish government has made a new proposition with the goal of 50% of newly recruited professors to be women ^6^. It is therefore of interest to see whether or not the same type of bias in *peer review* processes still exist in Sweden today.

Over the last years, Karolinska Institutet (KI) has announced yearly around 10 junior faculty positions (equivalent to an Assistant Professorship) with salary for four years. At KI, there is not yet a full tenure track system; once the four year faculty position is ended you have to apply for continued funding to stay in the academic career track. At each level, the competition gets harder and many researchers fall out of the system. At each level, there are un-proportionally more women that disappear, referred to as *the leaky pipeline.* This is illustrated by the number of assessed and granted faculty positions at KI from 2011–2014 ( Supplementary Figure 1). In 2011 and 2012, the proportion of assessed applications was equal between men and women, but not reflected by the proportion of granted applications; men had a higher success rate. For 2014, only applications passing the first bibliometric criteria were assessed (see *Methods* for details), women dropped out at an early stage and did not make it into the figure for comparison.

Thus, the aim of this investigation was to assess how applications submitted for Assistant Professorship positions at KI in 2014 were evaluated by *peer review* processes. A specific focus was made on diversity, where gender, ethnicity and academic age were among the variables studied. We further calculated a composite bibliometric score to analyze productivity among the applicants, and compared to the scores received by the external reviewers. In addition, an attempt to investigate whether influence from senior researchers at KI, research field, international experience and family situation mattered was made.

## Methods

### Description of applicants and the peer review process

The selection of applications for our study was based on the 2014 application process to become an Assistant Professor at KI. Eligibility criteria included a maximum academic age of seven years (number of years since PhD, excluding parental leave, clinical work or sick leave) and not having a permanent position at KI (e.g., technical staff or lab managers, which is often used as temporary solutions when postdocs cannot prolong their positions anymore). There were 150 applications submitted and 56 passed the first cut-off criteria of having a total journal impact factor of all publications >75 and were consequently sent for external review. The review panel consisted of six professors from other universities in Sweden (three men and three women). They were instructed to read the applications and score them based on 1) merits (publications and training) and 2) project plan (aim, novelty, methodology and feasibility). The scale ranged from 0-7 (0, insufficient; 1, bad; 2, weak; 3, good; 4, very good; 5, very good to excellent; 6, excellent; 7, outstanding). The total score of an application was the sum of both parts from all reviewers (maximum possible score on merits/project was 7 points * 6 reviewers = 42 and total was 2 * 42 = 84 for both parts), which gave a rank of the applicant in comparison to the other applicant´s scores. The applications were not blinded in any way and there was no information on how to be aware of, and deal with, biases from gender, ethnicity, age, etc. in the instructions sent to the reviewers.

### Assessed variables

The 56 applications read by the reviewers were assessed and discussed by both authors (SH and SH) according to different variables ( Table 1). Undergraduate education was grouped into the following categories: 1) medical, 2) engineering, 3) science, 4) other. Ethnicity was based on the reported “mother tongue”, and information on children was found in the CV or from time deducted from research due to parental leave. Funding was reported in the CV and the total amount was calculated and divided into own funding as principal investigator (PI) and as co-PI. If the amount received was missing, it was estimated based on type of funding (postdoc fellowship, small project grant, travel grant, etc.) in relation to what the other applicants reported. International experience was judged as having done education or research for at least six months at any University outside of Sweden. A high-rank University experience was judged as having done education or research at any of the 10 top-ranked Universities according to the QS World University Rankings®, 2014/15 ( Supplementary Table 1). Moreover, the number of supervised doctoral students as main or co-supervisor was counted. To be able to assess the KI network of the applicant, the number of women/men KI-affiliated references/instructors/mentors mentioned in the application was counted. The project plan submitted by the applicant was grouped into research field using the same division as done by the Swedish Research Council ( Supplementary Table 2) and categorized into method used ( Supplementary Table 3). Three applicants did not provide a project plan and were hence excluded from analysis wherever the project score was included.

**Table 1.** Characteristics of the applicants for Assistant Professorship positions at Karolinska Institutet 2014 (n=56).

**Continuous variables - mean ± SD**	
Scores received on merits	27.2 ± 4.3
Scores received on project plan	28.3 ± 3.6
Academic age - yrs	5.1 ± 1.5
Grants as PI, kSEK	2236 ± 3783
Grants as co-PI, kSEK	3635 ± 5808
Number of KI-affiliated Men	2.9 ± 2.8
Number of KI-affiliated Women	1.4 ± 1.5
H-index	9.0 ± 2.9
Total number of publications	20.4 ± 10.5
Publications, first author	6.6 ± 3.6
Publications, last author	0.7 ± 1.7
Total citations	397.9 ± 270.1
**Categorical variables – freq. (%)**	
Sex	
Women	21 (38)
Men	35 (62)
Ethnicity	
Swedish	24 (43)
European (except Swedish)	21 (38)
Asian	9 (16)
Other	2 (4)
Undergraduate degree	
Medical doctor	9 (16)
Engineer	8 (14)
Science	34 (61)
Other	5 (9)
Children	16 (29)
Main supervisor experience	5 (9)
Co-supervisor experience	34 (61)
International experience	48 (86)
High rank university experience	15 (27)
Published in high impact journal	13 (23)
Research field	
Biochemical structure and metabolism	2 (4)
Cancer	5 (9)
Cell and molecular biology	10 (18)
Developmental biology	1 (2)
Diabetes	1 (2)
Genetics	4 (7)
Microbiology, immunology and infectious diseases	8 (14)
Nervous system	11 (20)
Other	6 (11)
Pharmacy	2 (4)
Psychiatric diseases	3 (5)
Public health	2 (4)
Sensory organs	1 (2)

SD: standard deviation; PI: principal investigator; kSEK: thousands Swedish crowns.

### Bibliometric parameters of the applicants

The total number of publications and the number of first and last authorship positions were assessed from the publication list provided by the applicant. The number of high impact publications were defined as having lead authorship position (first or last) in any of the 30 top-ranked journals according to the Journal Citation Reports® 2014 ( Supplementary Table 4). Total number of citations was reported in the CV as well as the H-index ( *h*), which is defined as *h* number of publications with *h* number of citations. A composite bibliometric score was subsequently calculated corresponding to Wennerås & Wold ^2^ by summarizing standardized values of: 1) total number of citations, 2) total number of publications, 3) number of first authorship publications, 4) number of last authorship publications, 5) H-index, and 6) high impact publication (yes or no).

### Bibliometric parameters of the KI- affiliated researchers connected to the applicants

The effect of having a broad network at KI was assessed using bibliometric parameters as follows. The applicants were divided into four groups based on quartiles (Q1-4) of the scores received on merits by the external reviewers. All KI researchers connected to the respective applicant were consequently pooled in these four groups, and stratified by the source of connection to the applicant: 1) PhD-supervisor, 2) postdoc-supervisor, 3) collaborator, and 4) used as reference. By advice and help of the University Library at KI, bibliometric parameters for each researcher was derived from verified publications (articles and reviews) available between 1995–2014 and presented as 1) Avg Pub = Average of the number of publications, 2) Cf = Average of the field normalized citation scores where high values indicate that several publications were highly cited compared to publications in the same research area, 3) Avg Perc Cf = Average of the field normalized citation percentile for the department of the researcher, 4) Share Top 5% = Proportion of the field normalized publications that belong to the 5% most highly cited in the world, 5) Cnormalized = Average of the normalized citation scores based on year and document type, but not field type, 6) Avg JIF = Average of the journal impact factors for the department of the researcher, and 7) Avg JCf = Average of the journal field normalized citation scores for the department of the researcher. The field normalized indicator is not calculated if the group had less than 50 publications during the analyzed period because of instability. The normalization procedure compensates for different citation patterns due to research area, publication year and article type. The bibliometric numbers for all described variables were collapsed in the four groups as we were only allowed to present data on group level, hence, no statistical analyses were performed and only descriptive results were presented.

### Statistical analyses

All continuous variables were tested for normality and skewness, and log-transformed if skewed. Linear regression analysis was carried out in SAS 9.4 with PROC REG for each continuous variable as exposure, stratified by sex, with scores received on merits as outcome. The significance of the model was reported as trend. For binary variables, Fisher’s exact test was carried out using PROC FREQ on the different quartiles based on scores received on merits. Multivariable analysis was performed by step-wise regression using PROC PHREG procedure to scrutinize variables of importance for the outcome (scores received on merits) for different groups of applicants (men, women, Europeans, non-Europeans). The principal component analysis (PCA) used for pattern recognition analysis was done in the Soft Independent Modeling of Class Analogy (SIMCA 13, Umetrics®, Umeå, Sweden). The PCA is designed to extract and display the systemic variation in data sets and pre-process variables by scaling and mean centering in order to standardize weighting of each parameter. The first component in the PCA represents the largest variation in the data set, the second component the largest of the remaining variance, etc. The PCA creates a score plot showing the cluster of individuals in groups, and a loading plot identifying variables important for creating these clusters. The location of the individual in the score plot corresponds to the variable distribution in the loading plot. The PCA plots were re-generated using the plotly function in R for interactive online figures.

## Results

### Characteristics of the applicants

**Figure 1.**
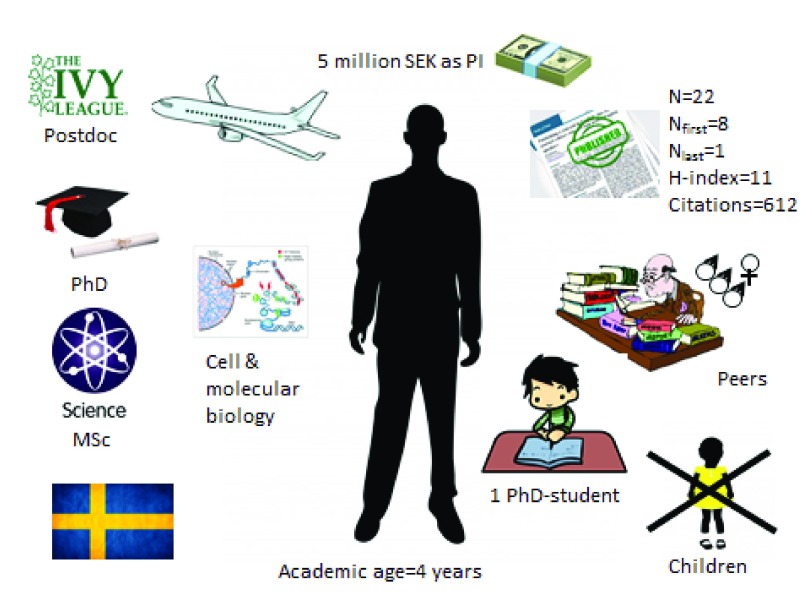
Characteristics of an average successful applicant for an Assistant Professorship position at Karolinska Institutet (KI). The average of an applicant in Q1, hence a successful applicant, is illustrated in the figure. In brief this person would be a Swedish man with a science degree and a PhD in cell and molecular biology. The person would have spent a postdoc abroad at one of the top 10 universities in the world, and has an academic age (the time since PhD) of about four years. The person has been successful in retrieving grants as principal investigator (PI) of about 5 million Swedish crowns, has published 22 research articles with eight as first author. Moreover, this person does also have a good network of peers at KI, mostly men, has one PhD-student of his own and no children so far.

The average applicant was a man from Sweden or another European country with some international experience, who co-supervised PhD-students and had received grants; both as PI and as co-PI. The average bibliometric variables were 20 published articles, seven as first and one as last author, with about 400 citations ( Table 1). In contrast, a successful top-ranked candidate, found in the first quartile (Q1) of the scores received on merits by the external reviewers, had received more funding, did their postdoc at high ranked Universities, supervised one PhD-student and published 22 articles ( Figure 1).

### Variables of importance for scores received on merits

**Figure 2.**
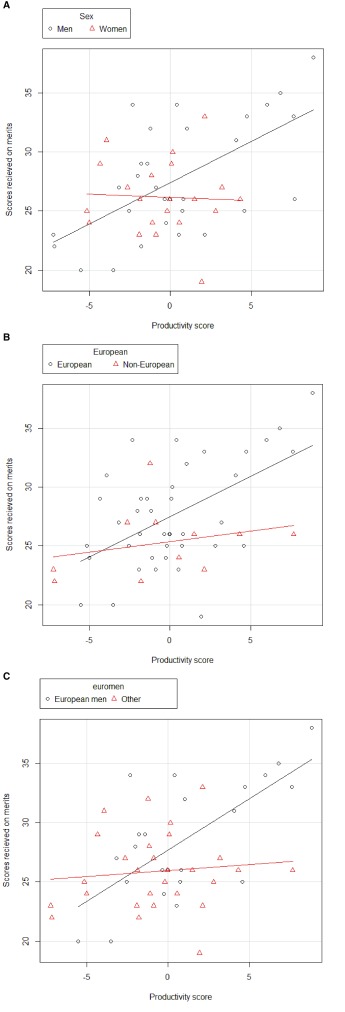
Scatterplot of the association between productivity and merits, grouped by sex, in applications for faculty position at Karolinska Institutet. A productivity score (x-axis) was calculated for each applicant by equal weights of the following bibliometric parameters: 1) total number of citations, 2) total number of publications, 3) number of first author publications, 4) number of last author publications, 5) H-index, and 6) high impact publication with lead author position (yes or no). On the y-axis, the scores received by the external reviewers on the merits of the applicant were plotted. ( **A**) For men, a clear association between productivity and merits was detected (P-value=0.0004). For women, on the contrary, there was no association found (P-value=0.84). ( **B**) For applicants who came from Europe originally, an association between productivity and merits was detected (P-value=0.0003), while there was no association found for non-Europeans (P-value=0.42). ( **C**) Finally, the combination of being male and from Europe was also found to have a strong association (P-value=0.0004), which was not seen in the other applicants (P-value=0.60).

To explore the impact of different variables on the success rate, data were divided into quartiles based on the scores received on merits by the external reviewers ( Table 2). Only two women were found in Q1, while the gender distributions in Q2-4 were almost equal. In men, univariate analysis revealed a positive association between scores received on merits and the composite bibliometric score (Trend test P-value=0.0004), while this was not true for women (P-value=0.84; Figure 2A). The association seen in men remained significant even after removing the top five applicants (data not visualised). Likewise, in Europeans, a positive association between scores received on merits and the composite bibliometric score was shown (P-value=0.0003), while not in non-Europeans (P-value=0.42; Figure 2B). The positive trend was also seen when comparing European men only (P-value=0.0004) to all other applicants (P-value=0.60; Figure 2C). Moreover, applicants with a background from the Middle East were un-proportionally found in the lowest quartile based on scores received on merits (Fisher’s exact test P-value=0.007). The benefit of having obtained grants was important for men, with an association with scores received on merits, as PI (P-value=0.03) and as co-PI (P-value=0.009). This was not true for women, although they obtained the same amount of funding overall. An international experience did not influence the score outcome unless it was spent at one of the top universities; border line significance was found in the Fisher’s exact test for top university experience grouped by scores on merits (P-value=0.058). There were no significant effects of having children or from the academic age on the score outcome, although both variables seemed to have an inverse correlation. Scores received on the project plan were significantly associated with scores received on merits, especially for women (P-value=0.0002), but also for men (P-value=0.045).

**Table 2.** Assessed variables stratified by sex and divided in quartiles based on scores received on merits.

Scores recieved on merits, quartiles	Q1 (38-31)	Q2 (30-27)	Q3 (26-25)	Q4 (24-19)	P-value	Correlation
Total N (56)	14	11	16	15		
**Continous variables - mean ± SD**						
Scores received on project plan						
Women	33.5 ± 2.1	29.2 ± 2.6	26.4 ± 2.4	25.2 ± 2.5	0.0002	0.73
Men	31.5 ± 3.5	29.8 ± 1.8	27.1 ± 4.1	26.1 ± 1.7	0.045	0.34
Composite bibliometric score						
Women	-0.92 ± 4.28	-0.79 ± 2.59	0.19 ± 3.12	-1.08 ± 2.36	0.84	-0.05
Men	3.58 ± 3.89	-1.86 ± 0.84	1.55 ± 3.43	-2.86 ± 3.56	0.0004	0.61
Academic age, yrs						
Women	4.5 ± 0.7	4.9 ± 1.2	5.5 ± 1.3	5.8 ± 1.6	0.2	-0.29
Men	4.5 ± 1.6	5.0 ± 0.7	5.2 ± 2.0	5.3 ± 1.8	0.5	-0.12
Grants as PI, kSEK						
Women	5631 ± 892	2379 ± 2414	1754 ± 1469	748 ± 667	0.58	0.13
Men	5282 ± 7002	1474 ± 484	581 ± 589	769 ± 570	0.03	0.36
Grants as co-PI, kSEK						
Women	0 ± 0	5793 ± 8195	6442 ± 8622	1983 ± 3073	0.38	-0.20
Men	5691 ± 4930	0 ± 0	4044 ± 7171	792 ± 1966	0.009	0.44
**Categorical variables – freq. (%)**						
Women	2 (14)	6 (55)	7 (44)	6 (40)	0.17	
International experience	12 (86)	10 (91)	13 (81)	13 (87)	0.96	
High rank university experience	7 (50)	2 (18)	5 (31)	1 (7)	0.058	
Children	2 (14)	4 (36)	4 (25)	7 (47)	0.26	
Swedish	8 (57)	4 (36)	6 (38)	6 (40)	0.7	
European	13 (93)	9 (82)	13 (81)	10 (67)	0.38	
Middle east	0 (0)	0 (0)	0 (0)	4 (27)	0.007	

SD: standard deviation; PI: principal investigator; kSEK: thousands Swedish crowns

In multivariate analysis, step wise regression was carried out in men and women separately to explore important factors for explaining the outcome. In men, the most contributing factors for a high score on the application was 1) the composite bibliometric score, 2) score based on project plan, and 3) grants as PI (all P-value<0.001). In women, the only variable that had any impact on outcome was score based on project plan (P-value=0.004).

### Distribution of KI- affiliated researchers connected to the applicants

The numbers of the KI-affiliated researchers for each quartile group of applicants were presented stratified on gender and the source of connection ( Table 3). For PhD-supervisors, the numbers were fairly constant across all quartiles, although there were more men (n=6) than women (n=1) in Q1. The postdoc-supervisors in Q1 were only two, possibly reflecting that most applicants in Q1 did not stay at KI during their postdoc training. The number of reference persons were also lower in Q1 overall, and had higher numbers for men in Q2 and Q3, while Q4 was even for both genders. When looking at collaborators, there was an interesting gender difference observed. Men were about twice as likely being collaborators in Q1-3, on almost constant levels, compared to women. However, in Q4 the opposite was true, in which women were twice as likely being collaborators than men. A general interpretation would be that applicants in Q4 were more likely to be connected to women researchers while the opposite was true for Q1.

**Table 3.** Bibliometry of the KI-affiliated researchers connected to the applicants.

Quartiles (Scores received on merits)	Q1 (38-31)		Q2 (30-27)		Q3 (26-25)		Q4 (24-19)	
Group Size	Men	Women	Men	Women	Men	Women	Men	Women
**PhD Supervisor**	6	1	5	3	4	2	4	3
**Postdoc supervisor**	1	1	7	3	13	4	4	6
**Reference**	7	5	13	3	22	4	10	11
**Collaborators**	34	19	34	13	40	13	8	22
Avg P (Publications)								
**PhD Supervisor**	115		122		74		205	
**Postdoc supervisor**	204		119		111		115	
**Reference**	118		100		106		132	
**Collaborators**	86		82		82		83	
**Total**	523		423		373		535	
Cf								
**PhD Supervisor**	1.5		2.1		1.5		1.8	
**Postdoc supervisor**	2.6		2.4		1.8		2.0	
**Reference**	2.0		1.9		1.6		1.7	
**Collaborators**	1.9		2.0		1.7		1.7	
**Total**	8		8.4		6.6		7.2	
Share Top 5%								
**PhD Supervisor**	11%		15%		8%		13%	
**Postdoc supervisor**	22%		15%		11%		13%	
**Reference**	16%		12%		10%		12%	
**Collaborators**	14%		12%		11%		11%	
**Total**	63%		54%		40%		49%	
Cnormalized								
**PhD Supervisor**	2.0		3.3		2.3		2.7	
**Postdoc supervisor**	3.6		4.1		2.6		3.3	
**Reference**	2.9		3.2		2.4		2.7	
**Collaborators**	2.9		3.0		2.6		2.7	
**Total**	11.4		13.6		9.9		11.4	

**groupsize** = Number of researchers within the cohort **P** = Number of verified Articles & Reviews during the analyzed timespan. Cf* = Average of the Field Normalized Citation Scores for verified Articles & Reviews. High values indicate that several publications are highly cited compared to publications in the same research area, however distribution may be highly skewed. **Share Top 5**%* = The proportion of publications that belong to the 5% most highly cited publications in the world (field normalized). High values indicate that many of the publications are among the world’s most highly cited publications within that field. **Cnormalized** = Average Normalized Citation Scores for verified Articles & Reviews. Normalization is done for publication year and document type, but not field type. Can be used in conjunction with Cf to distinguish effects of normalization of research area. *=Field normalized indicator. Is because of instability not calculated if the cohort has less than 50 publications during the analyzed period and it does not include publications published the current year -1. The normalization procedure compensates for different citation patterns due to research area, publication year and article type. **Certain data included herein were derived from the Web of Science ^®^ prepared by THOMSON REUTERS ^®^, Inc. (Thomson ^®^), Philadelphia, Pennsylvania, USA: © Copyright THOMSON REUTERS ^®^ 2015. All rights reserved.**

### Bibliometrics of KI- affiliated researchers connected to the applicants

The average number of publications per researcher was constant across Q´s with ^~^80 for the collaborators and ^~^100 for the other groups ( Table 3), with two exceptions; the PhD-supervisors in Q4 and the postdoc-supervisors in Q1 had about twice as many publications. The two postdoc-supervisors in Q1 published more than average, indicating that applicants in Q1 who stayed at KI chose successful researchers as supervisors. The same was true for the top 5% publications, where the Q1 group was generally better, especially considering the two postdoc-supervisors. However, in the totals of the field- and document normalized citation scores; Q2 out-performed the other groups, indicating that applicants in Q2 had a scientifically well performing network of researchers at KI, which were highly cited in their respective fields. The same pattern was seen in the normalized citation scores at departmental level, in which the Q2 group performed better than the Q1 group in two of three compared indicators ( Supplementary Table 5).

### Principal component analysis to identify clusters in data

**Figure 3.**
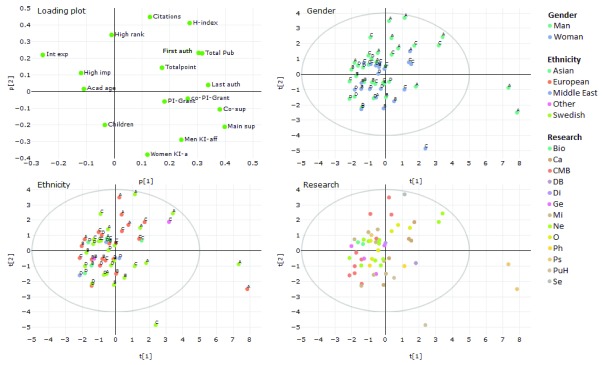
Principal Component Analysis (PCA) plots. The PCA is based on the variables assessed in applications for an Assistant Professorship position at Karolinska Institutet in 2014. The PCAs were created for a visible observation of the relationships between the variables and the scores received on merits by the external reviewers. The loading plot ( **A**) shows the distribution of the variables and the closer together the more related they are. The location of an applicant in a score plot ( **B**– **D**) corresponds to a high level in variables located at the same location in the loading plot and a low level in variables located at the opposite location through origo in the loading plot. The score plots show the location of the applicants in regard to ( **B**) and ethnicity ( **C**) in relation to the quartiles based on the score of the merits. The research field in relation to the method used in the project is seen in the last plot ( **D**). Abbreviations: A=quartile 1, B= quartile 2, C= quartile 3, D= quartile 4. Int Exp=International Experience, High Imp=High Impact publications, Acad age=Academic age (years from PhD defense), High Rank=Post doc visit at a high ranked university (see Supplementary Table 1), First Auth=First Author publications, Last Auth=Last Author publications, Total Pub=Total number of publications, PI-Grant=Grants received as Principal Investigator, co-PI Gran=Grants received as Co-Principal Investigator, Main sup=Experience as main supervisor, Co-Sup= Experience as co- supervisor, Men KI-aff=Number of Man KI-affiliated researchers associated with the applicants, Women KI-aff=Number of Woman KI-affiliated researchers associated with the applicants. The other abbreviations are found in Supplementary Table 2 and Supplementary Table 3. The online version of Figures 3B–D are interactive. Clicking a data point will highlight individuals that share that variable both within and across score plots. For example, clicking a ‘woman’ data point highlights all women within the Gender score plot and all individuals in the Ethnicity and Research score plots who are women. Double click to reset the plot.

The PCA was created for a visible observation of the relationships between the variables and the scores received on merits based on the characteristics of the applicants. The loading plot shows the distribution of the variables influencing the outcome of the merit scores ( Figure 3A). The position of an applicant in a score plot corresponds to a high level in variables located in the same position in the loading plot, and a low level in variables located in the opposite position through origo. The first principal component explained 23% of the variance, and the second 15%. The first score plot shows the location of the applicants stratified by gender ( Figure 3B) and the second by ethnicity ( Figure 3C) in relation to the quartiles based on the scores received on merits (A–D). A corresponds to Q1, B to Q2 and so on. The third score plot illustrates the research field in relation to the method used in the project ( Figure 3D).

In the first score plot ( Figure 3B), the applicants with the highest total points, Q1 (A), did not form a separate group but were mostly located in the upper right quadrant corresponding to high numbers in citations, h-index and first author publications. The applicants in Q2 (B) were located close to origo in the upper left quadrant corresponding to high impact publications and postdoc visits at high ranked universities, Q3 (C) were spread all over the plot and Q4 (D) were mostly located in the lower left quadrant corresponding to having children.

In the second score plot ( Figure 3C), Swedish applicants were not located in a specific square of the PCA. The same was almost true for European applicants with the exception of only one European applicant in the lower right square, corresponding to experience as supervisor and receiver of previous grants. Noteworthy, the three applicants from the Middle East were found in the lower left quadrant, opposite to the quadrant where the highest ranked applicants were found.

In the last score plot, Figure 3D, projects in the research field of cell and molecular biology were found everywhere, although the majority of the applicants from Q1 (A) either had projects or methods in the research field of cell and molecular biology. The most heterogenity of research fields were found in the left upper quadrant corresponding to high impact publications and postdoc visits at high ranked universities in the loading plot.

## Discussion

In this paper, we described the main characteristics of applicants for a junior faculty funded position at KI in 2014, and highlighted the desired variables for a successful candidate. We showed that men’s scores were positively associated with bibliometric measures and funding, which was not true for women. In addition, applicants with a Swedish or European background were more likely to receive higher scores.

The study is a thorough investigation of biases in *peer review* processes for junior faculty positions at KI. However, some limitations are warranted. The data were sub-selected from all the applications, because only one third of them were externally assessed when a triage system using a bibliometric cut-off was applied. Therefore, the reviewer bias observed may be more prominent as it has been shown that *peer review* is poor at discriminating between highly qualified applicants ^7^. Moreover, we did not have the possibility to explore differences in rating between different reviewers. Finally, because of the triage system, the sample size is small and power is limited. For some variables, data were missing, and therefore imputation was done where possible.

In society today, the knowledge of perception due to social background, education, ethnicity, gender, religion, profession and country of residence is increasing. In academia, the consensus around the meritocracy system and the objectivity of *peer review* is being challenged and unconscious bias training have become popular ^8, 9^. More studies emerge on this topic pointing at different flaws using *peer review*, both at individual reviewer level (commensuration bias ^10^) and between different reviewers ^11^. Still, more work needs to be done; the significant gender bias exists even though the National Institute of Health (USA) changed their review process ^12^. Already in 2008, the European Research Council (ERC) created a gender balance working group, but the systematic lower success rates for women remains ^13^. Since 1997, when Wennerås and Wold published their article about gender bias, the research climate has changed ^2^, but our study shows that gender bias in *peer review* processes in Sweden still exists, inflicting advancement in the academic career ladder for women. A data simulation of a corporate organization show that minor disadvantages at junior level were likely to become an impregnable lead at senior level ^14^. Hence, if women were in majority at a low level in an organization and were just slightly disadvantaged, they only represent one third on the highest level. This is in line with the scenario of the *leaky pipeline* of women in academia. We suggest that much of the leak is attributed to the gender discrimination in the *peer review* processes along the academic track. A side note is our observation of the skewed gender distribution among the KI-affiliated researchers associated with the applicants; in Q1-Q3 there were twice as many men, while the opposite was true in Q4. Notably, the observation is strikingly similar to the distribution of men and women professors (3:1).

Moreover, in 2014, faculty funding for Swedish universities resulted in an uneven distribution in which women scientists received 80 million SEK less per year than men ^15^. A research career system built on mobility and rapid and vast publishing tend to impair the outcome for women researchers ^1^, since women traditionally are more involved in family life. However, this seems to be more true in the early stages of the academic career ^16^, meanwhile women with children become more efficient and are suggested to achieve better results than women without a family ^17^. The PCA analysis demonstrated an inverse correlation between having children and scores received on merits, but we could not link this observation specifically to women in our analysis. However, a family often slows down the production speed, resulting in fewer publications ^18^, shorter postdoc visits abroad and a higher academic age, resulting in less funding and more time spent on getting alternative funding, as commissioned research on short time contracts. In the long run, the production is further slowed down and an independent research platform delayed. The uncertainty combined with the necessity for economic stability either encourages these women to take on positions as lecturers, or leaving academia - both resulting in the *leaky pipeline* and a reduced number of women professors.

Also the masculine stereotyping related to leadership positions is negative ^19^; the Swedish University of Agricultural Sciences concluded that qualified women did not think it was worth applying to a call for a professor launched in a way that only attracted men ^20^. Similarly, a recent study in *Science* showed that stereotyping in higher levels also extends to ethnic underrepresentation in academia ^21^, in line with our observation of Middle East applicants ended up un-proportionally in Q4.

In our bibliometric analysis of KI researchers connected to the applicants, the Q2 group had higher normalized citations scores, indicating well cited publications within their fields. Interestingly, Q2 was the only group with a majority of women applicants. It could be speculated that women applicants may have been higher scored if the quality of their publications had been assessed in field context. In other words, to overcome gender bias in publication rates, a shift from quantity to quality is warranted. Ingegerd Palmér, former Vice-Chancellor of Mälardalen University in Sweden, also concluded already in 2007 that women, despite fewer publications, were assessed equally to men in qualitative measures ^22^. A similar conclusion was made by a gold medalist in the Athena Swan, at University of York, UK, accredited for their work on gender equality ^23^. Women often reach the final evaluation process but are down prioritized when personal assessments of committee members are decisive. Researchers working in close collaboration with senior successful professors were referred to as “well-connected” if they were men, but “dependent” if they were women by committees at the Swedish Research Council ^24^. Hence, many women researchers get stuck in a vicious circle, facing a different trajectory in terms of advancing on the academic ladder than men at similar positions ^16^. In addition, women professors are reported to collaborate less with women at junior faculty positions compared to what male professors and male junior faculty do ^25^. However, women that do survive in academia eventually catch up with men in research output.

For future directions, direct feedback on present funding applications would improve future ones. We also suggest a transparent decision making process with gender neutral announcements of positions, mentor programs to develop networks for the non-normative applicants, as of a non-European ethnicity. To compensate for a slow production rate, we suggest additional merits for scientific competence; commitment in education, institutional citizenship (administrative and organizational work at departmental/university level) and the third objective should be rewarded.

To increase a gender and ethnicity neutral *peer review* process, we suggest a standard *peer review* based external assessments of blinded project descriptions and standardized automatic evaluations of merits and bibliometric, based on a composite productivity score.

To conclude, we demonstrate a positive bias for European men to be selected for faculty positions 2014 at KI after *peer review* evaluations. The successful candidate was a Swedish man without family with a thesis defense four years earlier, a high h-index, and a vast network of men researchers at KI. With the purpose to nurture ground-breaking and innovative research, we suggest multiple evaluation measures of young researchers to promote equality and diversity in academia.

## Data availability

The data referenced by this article are under copyright with the following copyright statement: Copyright: © 2018 Holst S and Hägg S

Data associated with the article are available under the terms of the Creative Commons Zero "No rights reserved" data waiver (CC0 1.0 Public domain dedication). 

The data used in this paper are based on public documents from Karolinska Institutet where the identity of the applicants have been kept anonymous in this paper and results presented in tables are based on group-level data only. In Sweden there is a law controlling all documents registered at a governmental agency, e.g., a university such as Karolinska Institutet, which says that they are open to the public (“Offentlighetsprincipen”). Hence, anyone can ask to get any document, such as applications for a position and instructions to reviewers, unless they are classified as secret. More information is available at http://ki.se/en/staff/official-documents-and-disclosure.

## Supplementary material

**Supplementary Figure 1: Distribution of assessed and granted Assistant Professorship positions at Karolinska Institutet 2011–2014 stratified by gender.** In 2011 and 2012, the proportion of assessed applications for a faculty position at Karolinska Institutet were almost equal between men and women; however, this was not reflected in the number of granted applications where men had a much higher success rate. In 2014, the proportions of assessed and granted applications are equal across gender. However, the number of assessed applications were only those passing the first bibliometric criteria (a total journal impact factor of all publications > 75), hence, more women most likely did not pass the first cut and did not make it into the assessment group in the figure.

Supplementary Table 1. Top-ranked Universities according to the QS World University Rankings ^®^, 2014/15 ^26^.

Supplementary Table 2. Research fields defined by the Swedish Research Council.

Supplementary Table 3. Method used in project plan.

Supplementary Table 4. Top-ranked journals according to the Journal Citation Reports ^®^ 2014 ^27^.

Supplementary Table 5. Bibliometry of departmental variables of the KI-affiliated researchers connected to the applicants.
