# Supplementary figures and images for: Positive bias for European men in peer reviewed applications for faculty position at Karolinska Institutet
Source: F1000Res. 2018 Aug 14;6:2145. Originally published 2017 Dec 18. [Version 2] doi: 10.12688/f1000research.13030.2 (PMC6092899; doi:10.12688/f1000research.13030.2)

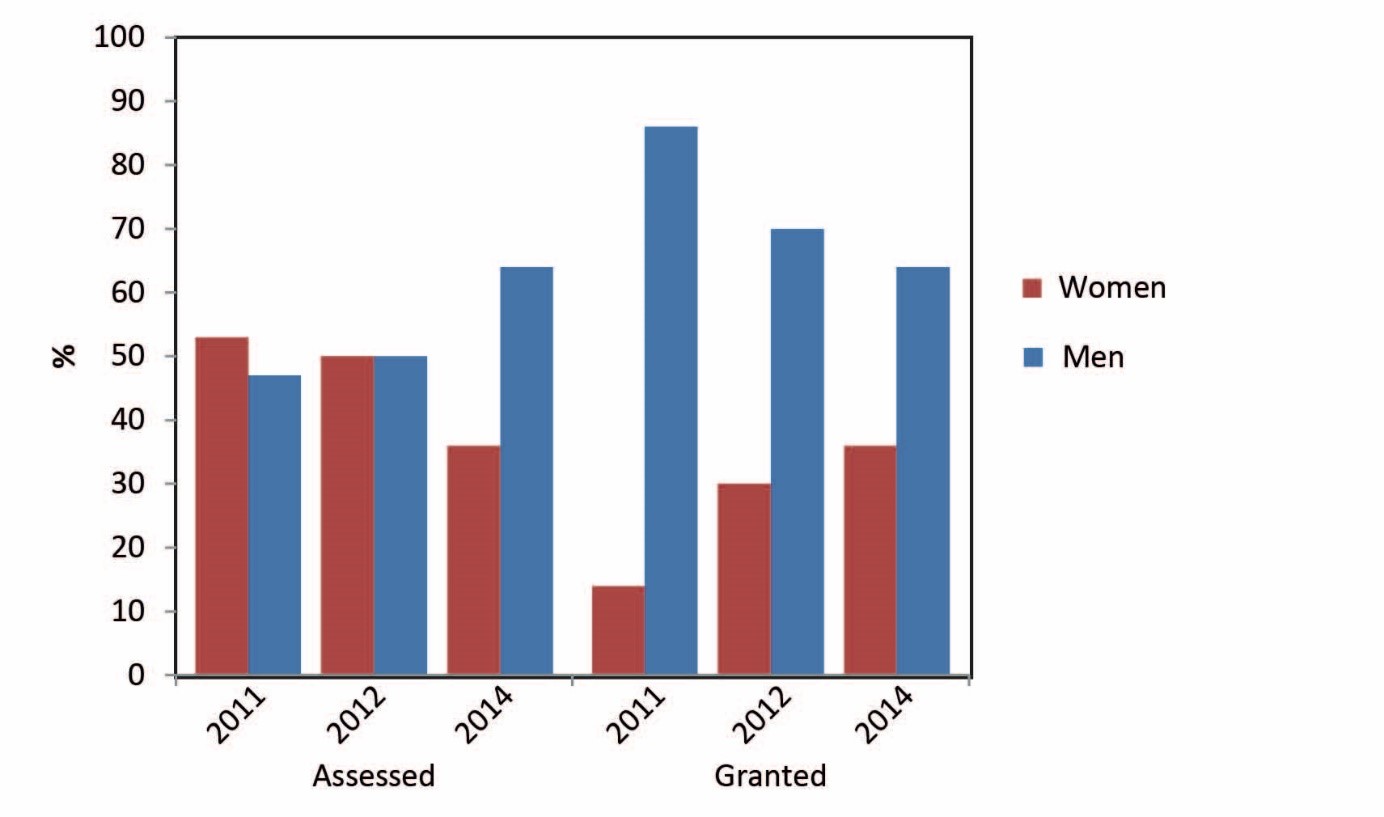

Supplement: Supplementary file 1 [file f1000research-6-17393-s0000.tgz › 599459f3-e421-4edd-be99-a4f9bb1ac824.jpg]
