# Supplementary material for: Positive bias for European men in peer reviewed applications for faculty position at Karolinska Institutet
Source: F1000Res. 2018 Aug 14;6:2145. Originally published 2017 Dec 18. [Version 2] doi: 10.12688/f1000research.13030.2 (PMC6092899; doi:10.12688/f1000research.13030.2)
Supplement: Supplementary file 2 [file f1000research-6-17393-s0001.tgz › e29893ea-c37e-4340-a714-10ad31b91d83.docx]

### Supplementary Table 1. Top-ranked Universities according to the QS World University Rankings ®, 2014/15

| 1 | Massachusetts Institute of Technology (MIT) |
| --- | --- |
| 2 | University of Cambridge |
| 3 | Imperial College London |
| 4 | Harvard University |
| 5 | University College London (UCL) |
| 6 | University of Oxford |
| 7 | Stanford University |
| 8 | California Institute of Technology (Caltech) |
| 9 | Princeton University |
| 10 | Yale University |
