# Supplementary material for: Positive bias for European men in peer reviewed applications for faculty position at Karolinska Institutet
Source: F1000Res. 2018 Aug 14;6:2145. Originally published 2017 Dec 18. [Version 2] doi: 10.12688/f1000research.13030.2 (PMC6092899; doi:10.12688/f1000research.13030.2)
Supplement: Supplementary file 3 [file f1000research-6-17393-s0002.tgz › 68d50227-db9b-461f-a2e1-8d575c3f618e.docx]

### Supplementary Table 2. Research fields defined by the Swedish Research Council

| Research field | Abbreviation |
| --- | --- |
| Anesthesiology and intensive care | An |
| Biochemical structure and metabolism | Bi |
| Cancer | Ca |
| Cell and molecular biology | CMB |
| Coagulation and thrombosis | Co |
| Dermatology | De |
| Developmental biology | DB |
| Diabetes | Di |
| Digestive system and kidney | DSK |
| Drug addiction | DA |
| Endocrinology | En |
| Environmental medicine and toxicology | ENT |
| Genetics | Ge |
| Heart and blood vessels | HBV |
| Microbiology, immunology and infectious diseases | Mi |
| Musculoskeletal system | MS |
| Nervous system | Ne |
| Nursing | Nu |
| Odontology | Od |
| Pharmacy | Ph |
| Prenatal and perinatal research | Pre |
| Psychiatric diseases | Ps |
| Public health | PuH |
| Radiology and imaging techniques | RIT |
| Reproductive system | Re |
| Respiratory system | RS |
| Sensory organs | Se |
| Other | O |
