# Supplementary material for: Positive bias for European men in peer reviewed applications for faculty position at Karolinska Institutet
Source: F1000Res. 2018 Aug 14;6:2145. Originally published 2017 Dec 18. [Version 2] doi: 10.12688/f1000research.13030.2 (PMC6092899; doi:10.12688/f1000research.13030.2)
Supplement: Supplementary file 4 [file f1000research-6-17393-s0003.tgz › 95396ebb-ebfb-437d-9c2b-99946f0bd49b.docx]

### Supplementary Table 3. Method used in project plan

| Cell and molecular biology on humans (CMH) |
| --- |
| Cell and molecular biology on animals (CMA) |
| Public Health (PuH) |
| Animal research (AR) |
| Clinical Research (CR) |
| Biotechnology (BT) |
| Other (O) |
| Not Applicable-project plan missing (NA) |
