# Supplementary material for: Positive bias for European men in peer reviewed applications for faculty position at Karolinska Institutet
Source: F1000Res. 2018 Aug 14;6:2145. Originally published 2017 Dec 18. [Version 2] doi: 10.12688/f1000research.13030.2 (PMC6092899; doi:10.12688/f1000research.13030.2)
Supplement: Supplementary file 5 [file f1000research-6-17393-s0004.tgz › 5b1d5ee1-f217-43c3-ac8d-b411a29d4fcb.docx]

### Supplementary Table 4. Top-ranked journals according to the Journal Citation Reports® 2014

| 1 | ANNU REV BIOCHEM |
| --- | --- |
| 2 | ANNU REV IMMUNOL |
| 3 | CA-CANCER J CLIN |
| 4 | CELL |
| 5 | CHEM REV |
| 6 | CHEM SOC REV |
| 7 | JAMA-J AM MED ASSOC |
| 8 | LANCET |
| 9 | LANCET ONCOL |
| 10 | NAT BIOTECHNOL |
| 11 | NAT GENET |
| 12 | NAT IMMUNOL |
| 13 | NAT MATER |
| 14 | NAT MED |
| 15 | NAT METHODS |
| 16 | NAT NANOTECHNOL |
| 17 | NAT PHOTONICS |
| 18 | NAT REV CANCER |
| 19 | NAT REV DRUG DISCOV |
| 20 | NAT REV GENET |
| 21 | NAT REV IMMUNOL |
| 22 | NAT REV MOL CELL BIO |
| 23 | NAT REV NEUROSCI |
| 24 | NATURE |
| 25 | NEW ENGL J MED |
| 26 | PHYSIOL REV |
| 27 | PROG MATER SCI |
| 28 | PROG POLYM SCI |
| 29 | REV MOD PHYS |
| 30 | SCIENCE |
