# Supplementary material for: Positive bias for European men in peer reviewed applications for faculty position at Karolinska Institutet
Source: F1000Res. 2018 Aug 14;6:2145. Originally published 2017 Dec 18. [Version 2] doi: 10.12688/f1000research.13030.2 (PMC6092899; doi:10.12688/f1000research.13030.2)
Supplement: Supplementary file 6 [file f1000research-6-17393-s0005.tgz › 529e299d-c1af-49c9-a1d9-6d19920ea933.docx]

Supplementary Table 5. Bibliometry of departmental variables of the KI-affiliated researchers connected to the applicants

| Quartiles (Scores received on merits) | | Q1 (38-31) | Q2 (30-27) | Q3 (26-25) | Q4 (24-19) |
| --- | --- | --- | --- | --- | --- |
| Avg Perc Cf |  |  |  |  |  |
| PhD Supervisor | | 0.64 | 0.66 | 0.63 | 0.59 |
| Postdoc supervisor | | 0.69 | 0.63 | 0.64 | 0.66 |
| Reference | | 0.66 | 0.64 | 0.63 | 0.62 |
| Collaborators | | 0.65 | 0.62 | 0.62 | 0.62 |
| Total |  | 2.64 | 2.55 | 2.52 | 2.49 |
| Avg JIF |  |  |  |  |  |
| PhD Supervisor | | 4.7 | 7.1 | 6.0 | 5.5 |
| Postdoc supervisor | | 7.3 | 8.4 | 6.2 | 7.3 |
| Reference | | 5.8 | 7.7 | 5.9 | 5.9 |
| Collaborators | | 6.3 | 6.6 | 5.9 | 6.2 |
| Total |  | 24.1 | 29.8 | 24 | 24.9 |
| Avg JCf |  |  |  |  |  |
| PhD Supervisor | | 1.3 | 2.0 | 1.5 | 1.5 |
| Postdoc supervisor | | 2.0 | 2.1 | 1.7 | 1.8 |
| Reference | | 1.6 | 1.9 | 1.6 | 1.5 |
| Collaborators | | 1.7 | 1.8 | 1.6 | 1.6 |
| Total | | 6.6 | 7.8 | 6.4 | 6.4 |

**Avg Perc Cf*** = The average Field Normalized Citation Percentile for department’s verified Articles & Reviews. High values indicate that many of the publications are highly or very highly cited within that research area. This indicator is less sensitive than Cf to extreme citation values for individual publications.

**Avg JIF** = Average of the Journal Impact Factors for the department’s verified Articles & Reviews. High values indicate that many of the publications are in journals that on average are highly cited, however distribution may be highly skewed.

**Avg JCf*** = Average of the Journal Field Normalized Citation Scores for the department’s verified Articles & Reviews. High values indicate that many the publications are in field specific journals that on average are highly cited within that field, however distribution may be highly skewed.

*****=Field normalized indicator. Is because of instability not calculated if the cohort has less than 50 publications during the analyzed period and it does not include publications published the current year -1. The normalization procedure compensates for different citation patterns due to research area, publication year and article type.

**Certain data included herein were derived from the Web of Science® prepared by THOMSON REUTERS®, Inc. (Thomson®), Philadelphia, Pennsylvania, USA: © Copyright THOMSON REUTERS® 2015. All rights reserved.**
