# Supplementary material for: Positive bias for European men in peer reviewed applications for faculty position at Karolinska Institutet
Source: F1000Res. 2018 Aug 14;6:2145. Originally published 2017 Dec 18. [Version 2] doi: 10.12688/f1000research.13030.2 (PMC6092899; doi:10.12688/f1000research.13030.2)
Supplement: Peer review history

# Peer review: Referee response for version 2

**DOI:** 10.5256/f1000research.17393.r37193

**Published:** 2018-08-14

**Authors:** Stephen A. Gallo

**Affiliations:** 1 Scientific Peer Advisory and Review Services (SPARS), American Institute of Biological Sciences (AIBS), McLean, VA, USA

Thank you for the updates. You have addressed my concerns.

I have read this submission. I believe that I have an appropriate level of expertise to confirm that it is of an acceptable scientific standard.

# Peer review: Referee response for version 1

**DOI:** 10.5256/f1000research.14128.r34299

**Published:** 2018-06-11

**Authors:** Stephen A. Gallo

**Affiliations:** 1 Scientific Peer Advisory and Review Services (SPARS), American Institute of Biological Sciences (AIBS), McLean, VA, USA

This manuscript pertains to a study in how applicants for faculty positions at Karolinska Institute (KI) are assessed. Applicants submit proposal information which is first passed through a Phase 1 cut-off whereby the applicants have to have published a requisite number of publications above a threshold impact factor to qualify for further review (roughly a third). Phase 2 is then an external review by a panel of 6 reviewers (3 male/ 3 female) outside of KI. The proposals were scored in two dimensions, once based on the merits of the applicants track records and training (Merits) and once on the quality of the research proposed (Project Plan). Scores from these two dimensions were added together to create the ranking. Demographic data was gleaned from applicant CVs, as was their publication list and any affiliated KI mentors/collaborators, etc. The bibliometric data from KI collaborators was gathered with the help of the University Library at KI. External reviewer Merit scores were plotted against the author’s productivity scores for different subsets of data (gender and ethnicity). Also Merit scores were separated into Quartiles (Q1-4), and proportionality across different variables was observed. Scores on Project plan were not analyzed much in this work, but were found to be well correlated with Merit scores for both men and women. Multivariate analysis was conducted as well as principal component analysis (PCA) to see if there is clustering.

 The applicants find substantial differences in distribution across Merit Score Quartiles between male/female applicants, as a disproportionate # of men appeared in Q1. Gender proportions were more balanced in other quartiles. Regression analysis revealed a significant relationship between Merit score and productivity scores for men, but not for women or for non-europeans. Step wise regression revealed productivity scores, Project Plan scores and the presence of grants all had significant relationships to Merit scores for men, but only Project Plan scores were important for women. Applicants in Q1 also were more likely to be connected to male collaborators than women. PCA suggested having children was somewhat associated with lower Merit scores (Q4) and citation levels and 1 ^st^ author publications were somewhat associated with Q1 Merit scores.  

 The goals of this research and the statistical analysis are straightforward, and there are some clear observations of not only disproportionate representation in grading but also review panels differentially emphasizing criteria across gender and ethnicity, specifically with bibliometric productivity and presence of grants. These results are disturbing as they suggest that biases are contributing to the observed disproportionate scoring. However, there are some issues that may need some clarification, consideration:

 Firstly, in Supplementary Fig 1, the proportion of women granted in 2011 and 2012 seem to be worse than 2014; as 2014 has a triage of sorts based on bibliometric productivity, does this mean that the current system (2014) is less biased than previous years? It seems as the proportions of women who applied vs granted for 2014 are pretty comparable, despite disproportionate representation in Q1 (Merit score). What are the reasons for this? Do Project scores compensate for biased Merit scores to push these applicants into the funding range? Looks like 38% of the total granted were women, which means about 4 out of the 10 granted were women. If only 2 women were granted from Q1 (merit score), but apparently 4 women were funded, 2 must have come from Q2-4, yet there were 12 other males in Q1. So either some males in Q1 merit score did not do well in their project scores, or the granting is not in strict order of rank? It would be interesting to know how the Project scores affected the ranking. Perhaps this could be addressed in the text.

 Also, in 2014, because of the triage, the review panel only evaluates a subset of already excellent applicants (based on bibliometrics). But peer review is known to be poor at discriminating between highly qualified applicants ^1^. This should probably be referenced and discussed in the text, as reviewer biases may be more prevalent in this situation.

 Secondly, in Fig 2, while male Merit scores correlated to productivity measurements, females scores did not. Yet, the authors mention that “The PCA analysis demonstrated an inverse correlation between having children and scores received on merits, as a family often slows down the production speed, resulting in fewer publications, shorter postdoc visits abroad and a higher academic age, resulting in less funding and more time spent on getting alternative funding, as commissioned research on short time contracts.” If female scores are not derived by the reviewers from their productivity, why would having children, and its effects on productivity, matter for reviewer’s scores? In fact, based on the regression, the authors state that “There were no significant effects of having children…on the score outcome.” So it’s a bit confusing what is happening here. Also, the authors mention women may be more affected by having children, “since women traditionally are more involved in family life.” Do the data show that having children and gender correlated in this sample?

 Thirdly, it is clear there are differences in how reviewers evaluate applicants of different gender. The authors may mention work by Carole lee on commensuration bias in the text, which I believe predicts this kind of behaviour ^2^. Out of curiosity, do the authors have any information about the reviewer discussions that could shed light to how the panel weighed criteria relative to applicant demographics? Also, some research has come out suggesting there is more variation across reviewers than across proposals ^3^. Do the authors have any information on how individual reviewer scores varied? Were some panelists more biased than others? Did this vary at all by reviewer gender? This may be beyond the scope of this study, but it might be appropriate to mention that there may be different sources of the bias, at the panel level vs individual level.

 A few more minor points:

 For the linear regressions, only p-values were reported in what the authors refer to as trend test. Could the authors include the correlation coefficient as well, as it seems there is a lot of spread in the data. Also, for Fig. 2c, the data for European men still have a good deal of variability that seems independent of actual productivity. Could the authors comment on potential sources for this variability?

 In the text, it is said that “information on children was found in the CV or from time deducted from research due to parental leave;” is this information always reported on a CV? It was mentioned the authors imputed missing data; did this include data on children?

 Citations are time and field dependent; were they normalized for this productivity measurement for the applicants? If not, it may be difficult to compare. It seems, though, that citations were normalized for the KI collaborators/mentors. It’s unclear why different bibliometric approaches were used for applicants vs collaborators. Also, h-index is sensitive to age, was there an attempt to account for this confounder?

I have read this submission. I believe that I have an appropriate level of expertise to confirm that it is of an acceptable scientific standard, however I have significant reservations, as outlined above.

## References

[1] van den BesselaarPSandströmU: Early career grants, performance, and careers: A study on predictive validity of grant decisions. *Journal of Informetrics*.2015;9(4) : 10.1016/j.joi.2015.07.011 826-838 10.1016/j.joi.2015.07.011

[2] LeeC: Commensuration Bias in Peer Review. *Philosophy of Science*.2015;82(5) : 10.1086/683652 1272-1283 10.1086/683652

[3] PierELBrauerMFilutAKaatzARaclawJNathanMJFordCECarnesM: Low agreement among reviewers evaluating the same NIH grant applications. *Proc Natl Acad Sci U S A*.2018;115(12) : 10.1073/pnas.1714379115 2952-2957 10.1073/pnas.1714379115 29507248PMC5866547

# Response

**Published:** 2018-08-09

**Authors:** Sara Hägg

**Affiliations:** Karolinska Institutet, Sweden

**Responses to reviewer comments by Stephen A Gallo on Aug 9th 2018:**

Firstly, in Supplementary Fig 1, the proportion of women granted in 2011 and 2012 seem to be worse than 2014; as 2014 has a triage of sorts based on bibliometric productivity, does this mean that the current system (2014) is less biased than previous years? It seems as the proportions of women who applied vs granted for 2014 are pretty comparable, despite disproportionate representation in Q1 (Merit score). What are the reasons for this? Do Project scores compensate for biased Merit scores to push these applicants into the funding range? Looks like 38% of the total granted were women, which means about 4 out of the 10 granted were women. If only 2 women were granted from Q1 (merit score), but apparently 4 women were funded, 2 must have come from Q2-4, yet there were 12 other males in Q1. So either some males in Q1 merit score did not do well in their project scores, or the granting is not in strict order of rank? It would be interesting to know how the Project scores affected the ranking. Perhaps this could be addressed in the text.

**Authors reply: We thank the reviewer for raising this concern and giving us the opportunity to clarify. It is true that in the graph provided in Supplementary Figure 1, the proportion of granted women were lower in 2011 and 2012 comparted to 2014. However, roughly half of the applicants are usually women, but in 2014 – because of the triage system used this year – the figure only represents applicants who passed the first cut-off, hence less women appear in the graph in 2014 in the “Assessed” category. That said, the proportion of assessed and granted women applicants (36%) were equal in 2014 after the triage was taken into account, but should perhaps have been 50% to be completely fair given that this was probably the proportion of women applicants before the triage was applied.**

**Nevertheless, since there were only 2 women in Q1 and 4 women who were granted the position, 2 women were taken from the Q2 category to be prioritized above men in Q1. This was done based on interviews of the candidates, the project plan had nothing to do with it. Most probably, the KI leadership decided to rank these two women higher in order to reach the same proportions in the assessed and granted categories of the applicants. Hence, KI are fully aware of the gender inequality situation and usually interviews 2 candidates for every position in order to have some freedom in whoever is chosen.**

Also, in 2014, because of the triage, the review panel only evaluates a subset of already excellent applicants (based on bibliometrics). But peer review is known to be poor at discriminating between highly qualified applicants ^1^. This should probably be referenced and discussed in the text, as reviewer biases may be more prevalent in this situation.

**Authors reply: We thank the reviewer for this comment. It is an interesting notation that reviewer bias may be more prominent because of the selection procedure done on the applications. Yet another reason for not conducting a bibliometric cut-off. We have added a sentence about this in the new version of the manuscript.**

***“*** ***The data were sub-selected from all the applications, because only one third of them were externally assessed when a triage system using a bibliometric cut-off was applied. Therefore, the reviewer bias observed may be more prominent as it has been shown that peer review is poor at discriminating between highly qualified applicants (*** ***van den Besselaar, 2015).*** **“**

Secondly, in Fig 2, while male Merit scores correlated to productivity measurements, females scores did not. Yet, the authors mention that “The PCA analysis demonstrated an inverse correlation between having children and scores received on merits, as a family often slows down the production speed, resulting in fewer publications, shorter postdoc visits abroad and a higher academic age, resulting in less funding and more time spent on getting alternative funding, as commissioned research on short time contracts.” If female scores are not derived by the reviewers from their productivity, why would having children, and its effects on productivity, matter for reviewer’s scores? In fact, based on the regression, the authors state that “There were no significant effects of having children…on the score outcome.” So it’s a bit confusing what is happening here. Also, the authors mention women may be more affected by having children, “since women traditionally are more involved in family life.” Do the data show that having children and gender correlated in this sample?

**Authors reply: We agree with the reviewer on the fact that we were not able to show any clear associations between having children and productivity score in our data. However, we could see an inverse association – less applicants with children in Q1 compared to Q4 – although the trend was not statistically significant. In the discussion section we try to highlight what is known around this topic, and we do not actually claim that we have seen a clear relationship between gender and having children in our data. The family situation may have impact on mothers as well as fathers on their productivity. To clarify, we modified the text in the discussion slightly.**

***“*** ***The PCA analysis demonstrated an inverse correlation between having children and scores received on merits, but we could not link this observation specifically to women in our analysis.*** ***“***

Thirdly, it is clear there are differences in how reviewers evaluate applicants of different gender. The authors may mention work by Carole lee on commensuration bias in the text, which I believe predicts this kind of behaviour ^2^. Out of curiosity, do the authors have any information about the reviewer discussions that could shed light to how the panel weighed criteria relative to applicant demographics? Also, some research has come out suggesting there is more variation across reviewers than across proposals ^3^. Do the authors have any information on how individual reviewer scores varied? Were some panelists more biased than others? Did this vary at all by reviewer gender? This may be beyond the scope of this study, but it might be appropriate to mention that there may be different sources of the bias, at the panel level vs individual level.

**Authors reply: We thank the reviewer for the additional suggestions. We have now added some text discussing commensuration bias and on variability in reviewer scores to the new version of the manuscript. Unfortunately, we do not have any other information regarding the differences in scoring that may have been observed between the different reviewers on the applications in our analysis. Moreover, there were no discussions, each reviewer submitted their scoring independently and the overall rating was done by the KI leadership. We have now added a sentence in the limitation section about this.**

***“*** ***More studies emerge on this topic pointing at different flaws using peer review, both at individual reviewer level (commensuration bias [Lee, 2015]) and between different reviewers [Pier, 2018].”***

***“*** ***Moreover, we did not have the possibility to explore differences in rating between different reviewers.”***

A few more minor points:

For the linear regressions, only p-values were reported in what the authors refer to as trend test. Could the authors include the correlation coefficient as well, as it seems there is a lot of spread in the data. Also, for Fig. 2c, the data for European men still have a good deal of variability that seems independent of actual productivity. Could the authors comment on potential sources for this variability?

**Authors reply: We thank the reviewer for posing these suggestions and improving the manuscript. We have now added a new column to table 2 where pearson correlation coefficients have been added adjacent to the trend p-values.**

**Regarding the second question, we agree on the fact that European men still have a great deal of variability explained by other factors than productivity. We ran a step wise regression in those 23 individuals with complete data and found the significant contributing factors to be:**

1. **Grants as PI (P-value=0.0004)**
2. **Grants as co-PI (P-value=0.0012)**
3. **Scores received on project plan (P-value=0.021)**
4. **Composite bibliometric score (P-value=0.0499)**

In the text, it is said that “information on children was found in the CV or from time deducted from research due to parental leave;” is this information always reported on a CV? It was mentioned the authors imputed missing data; did this include data on children?

**Authors reply: Information on children was not always present and we did not impute this variable. Hence, it is possible that there may be missing information regarding this variable that we cannot compensate for.**

Citations are time and field dependent; were they normalized for this productivity measurement for the applicants? If not, it may be difficult to compare. It seems, though, that citations were normalized for the KI collaborators/mentors. It’s unclear why different bibliometric approaches were used for applicants vs collaborators. Also, h-index is sensitive to age, was there an attempt to account for this confounder?

**Authors reply: We are aware of the fact that H-index is age sensitive and that citations may have been better used in normalized versions. However, these were the variables available in the CV and made available to the reviewers. Although it would have been interesting to investigate other bibliometric variables, this was not possible as we were restricted to use the variables provided by the applicants themselves. As for the KI collaborators, we could perform a deeper analysis using field normalized scores presented on group level with the help of the KI library, and only because these researchers were already KI affiliated.**

# Peer review: Referee response for version 1

**DOI:** 10.5256/f1000research.14128.r33112

**Published:** 2018-05-08

**Authors:** Inés Sánchez de Madariaga

**Affiliations:** 1 Technical University of Madrid, Madrid, Spain

The article is well written, structured and argued. The methodology used is appropriate and well applied. Bibliography, notes, and references to the state of the art appropriate. The article addresses an important issue regarding gender bias in the evaluation of scientific research on which analysis of empirical evidence is still scarce. I fully recommend its indexing.

I have read this submission. I believe that I have an appropriate level of expertise to confirm that it is of an acceptable scientific standard.

# Response

**Published:** 2018-05-17

**Authors:** Sara Hägg

**Affiliations:** Karolinska Institutet, Sweden

We thank the reviewer for the comments.
